# A Homozygous Missense Variant in *DCLK2* as a Potential Modifier of Disease Severity in *PDHA1*-Associated Primary Pyruvate Dehydrogenase Complex Deficiency

**DOI:** 10.3390/jcm15218336

**Published:** 2026-10-26

**Authors:** Dalal A. Al-Mutairi, Fahad K. Al-Mutairi, Athbi A. Naief, Trevor F. Batten

**Affiliations:** 1 Human Genetics Unit, Faculty of Medicine, Kuwait University, Kuwait City 13110, Kuwait; 2 Pediatric Department, Al- Jahra Hospital, Al-Jahra 00042, Kuwait; 3 Department of Radiology, Shaikh Jaber Al-Ahmad Al-Sabah Hospital, Kuwait City 47760, Kuwait; 4 Leeds Institute of Genetics and Rare Diseases, School of Medicine, University of Leeds, Leeds LS2 9JT, UK

**Keywords:** primary pyruvate dehydrogenase complex deficiency, Leigh syndrome, *PDHA1*, *DCLK2*, consanguinity, lactic acidosis, neuromuscular degeneration, ataxia, ophthalmoplegia

## Abstract

**Introduction:** Primary pyruvate dehydrogenase complex deficiency (PDCD) is a genetically heterogeneous mitochondrial disorder with marked clinical variability. *PDHA1* is the most common cause of PDCD, but additional genetic factors may contribute to disease severity. **Methods:** A consanguineous family with two affected male siblings with clinical features of PDCD underwent clinical and genetic investigation. Whole-exome sequencing (WES), X-chromosome analysis, genome-wide autozygosity mapping, and segregation analysis were performed. Clinical, biochemical, neuroimaging, ophthalmological, and longitudinal medical records were reviewed. In silico analyses were performed for the identified variants. **Results:** Both siblings presented with neonatal lactic acidosis and severe neurological disease, including Leigh brain abnormalities, followed by progressive neuromuscular and multisystem manifestations. WES identified a novel hemizygous *PDHA1* missense variant (c.754C>G; p.Leu252Val; rs1555934383), consistent with X-linked inheritance and providing the primary genetic explanation for PDCD. Autozygosity mapping identified a shared identical-by-descent interval on chromosome 4, within which a novel homozygous *DCLK2* missense variant (c.101G>A; p.Ser34Asn) was identified. The variant was present in both affected siblings and segregated within the family. Despite sharing both variants, the siblings showed marked differences in disease severity. The younger sibling developed severe neurological and respiratory complications and died in childhood following a surgical procedure, whereas the older sibling survived into late adolescence but developed substantial neurological, metabolic, respiratory, and ophthalmological complications, including profound visual impairment and bilateral optic atrophy. **Conclusions:** The *PDHA1* variant represents the primary genetic cause of PDCD, while the co-segregating homozygous *DCLK2* variant is a candidate modifier of disease severity. Its localization within the shared IBD interval and biological relevance to neuronal stress responses support this hypothesis and suggest that *DCLK2* may contribute to the phenotypic variability and severity of *PDHA1*-associated disease.

## 1. Introduction

Primary pyruvate dehydrogenase complex deficiency (PDCD) is a clinically and genetically heterogeneous mitochondrial disorder caused by impaired pyruvate oxidation and consequent reduction in cellular energy production [1,2]. The pyruvate dehydrogenase E1 component is an α_2_β_2_ heterotetramer composed of two E1α and two E1β subunits, with the E1α subunit encoded by *PDHA1* [1]. X-linked *PDHA1*-related E1α deficiency (PDHAD; OMIM 312170) is the molecular form identified in the present family [2,3].

The major genetic forms of PDCD include E1α deficiency (PDHAD; OMIM 312170), caused by X-linked *PDHA1* variants; E2 deficiency (PDHDD; OMIM 245348), caused by *DLAT* variants; E3-binding protein deficiency (PDHXD; OMIM 245349), caused by *PDHX* variants; E1β deficiency (PDHBD; OMIM 614111), caused by biallelic *PDHB* variants; and pyruvate dehydrogenase phosphatase deficiency (PDHPD; OMIM 608782), caused by *PDP1* variants [2,3]. Clinical manifestations are predominantly neurological and may include congenital microcephaly, epilepsy, ataxia, hypotonia, developmental delay, and structural brain abnormalities such as Leigh syndrome and/or dysgenesis of the corpus callosum. Principal metabolic abnormalities include lactic acidemia, increased plasma pyruvate, and metabolic acidosis [1,2].

In general, primary PDCD is diagnosed at a mean age of approximately 4 years, with a median age of around 1.5 years [4,5]. However, the disorder may be recognized prenatally when routine ultrasonography demonstrates abnormalities such as microcephaly, cerebellar hypoplasia, ventriculomegaly, paraventricular pseudocysts, delayed gyration, and/or dysgenesis of the corpus callosum. Fetal brain magnetic resonance imaging (MRI) may provide additional evidence, including cerebral volume loss and periventricular T2-weighted hyperintensity [6,7]. Newborns may present with fetal growth restriction, microcephaly, structural or functional abnormalities of the brain, and lactic acidosis [4,5]. Less commonly, PDCD may present later in childhood with episodic limb paralysis or alternating hemiplegia [8], paroxysmal dystonia or dyskinesia [9,10,11], or intermittent ataxia [5].

Developmental delay and axial hypotonia represent the most frequent neurological manifestations of PDCD. Other neurological features include ataxia, spasticity, epilepsy, and appendicular hypertonia [5], peripheral neuropathy [9], and episodes of dystonia triggered by stress, fever, or exercise [10,12]. Rare presentations include exercise-induced dyskinesia and episodic limb paralysis or hemiplegia [11]. Characteristic facial features may include a thin upper lip, long philtrum, and low-set ears [13,14]. Ophthalmological manifestations are uncommon and include optic atrophy, nystagmus, strabismus, ptosis [14], and ophthalmoplegia [8,14]. Growth abnormalities include intrauterine growth restriction and primary or acquired microcephaly, whereas shortening of the long bones is reported less frequently. Musculoskeletal abnormalities may include acquired hip dysplasia. Psychiatric manifestations, including auditory hallucinations and delusional thoughts, have also been described, particularly among mildly affected individuals during adolescence or early adulthood [1].

Common brain MRI abnormalities include cerebral atrophy, asymmetric ventriculomegaly, and dysgenesis or agenesis of the corpus callosum [9,14]. T2-weighted hyperintensities may also occur, most frequently involving the basal ganglia and, to a lesser extent, the brainstem and cerebellum [14]. Additional reported findings include periventricular cysts and/or intraventricular septations [15]. Hyporotation of the hippocampus is an uncommon MRI finding that has also been described in individuals with PDCD [5,15].

The principal laboratory abnormalities in PDCD are elevated blood lactate and pyruvate concentrations, generally with a lactate-to-pyruvate ratio within the normal range of approximately 10–20. Plasma pyruvate concentrations may exceed 0.2 mmol/L [16]. Lactate and pyruvate elevations may be intermittent and do not necessarily reflect disease severity, as pyruvate measurements can be affected by inappropriate sample collection and handling. Increased plasma proline and alanine concentrations have also been reported. Although the proline-to-leucine and alanine-to-leucine ratios may be sensitive indicators of primary PDCD, they are not specific to the disorder [17]. Increased lactate and pyruvate concentrations may also be detected in plasma, urine, and cerebrospinal fluid (CSF). Pyruvate dehydrogenase complex (PDC) activity may be markedly reduced in cultured lymphocytes, fibroblasts, and/or skeletal muscle, although enzyme activity can differ between tissues within the same individual [18]. Newborn screening for PDCD is not yet widely available. In a pilot study using dried blood spots, screening based on a proline-to-leucine ratio > 3 and/or an alanine-to-leucine ratio > 4 identified three neonates with PDCD and two additional neonates with other mitochondrial disorders. The screening approach had a positive predictive value of 2.8% and a sensitivity of 80% [17].

Among the genes associated with primary PDCD, *PDHA1* is the most frequently implicated, accounting for approximately 76–85% of cases. The *PDHA1* gene encodes the E1-alpha subunit of the pyruvate dehydrogenase complex and is inherited in an X-linked manner. Pathogenic *PDHA1* variants are associated with a broad clinical spectrum, ranging from neonatal lactic acidosis and Leigh syndrome [15,19,20] to later-onset manifestations such as myopathy and ataxia [21,22]. Female heterozygotes may also exhibit substantial and asymmetric brain MRI abnormalities, including ventriculomegaly and dysgenesis of the corpus callosum [18,23]. Although a ketogenic diet may improve the clinical course in some individuals, treatment response is variable, and prolonged survival has been reported in selected patients [15,24].

Doublecortin-like kinase 2 (*DCLK2*) encodes a serine/threonine protein kinase enriched in the brain, with established roles in neuronal development, survival, and cytoskeletal regulation. Increasing evidence also implicates *DCLK2* in neuronal responses to injury, particularly under ischemic and metabolic stress. Experimental studies of chronic cerebral ischemia and oxygen-glucose deprivation have demonstrated altered *DCLK2* expression in hippocampal neurons, while modulation of *DCLK2* expression influences neuronal survival [25,26,27]. Mechanistic studies further implicate *DCLK2* in neuronal inflammatory and cell-death pathways, including phosphorylation-dependent regulation of EHF and downstream activation of caspase- and GSDME-related pathways [28,29,30]. These pathways regulate neuronal survival and injury responses and may therefore be relevant to the severe neurological and visual manifestations observed in the present family, particularly the extensive visual pathway involvement in the affected individual.

In addition, the RNA-binding protein CPEB4 regulates neuronal gene expression and has an important role in cellular responses to metabolic and ischemic stress. Under ischemic conditions, CPEB4 increases *DCLK2* mRNA stability and expression, linking CPEB4-mediated post-transcriptional regulation to *DCLK2*-dependent neuronal stress responses [31,32,33,34]. Although these findings do not establish a role for *DCLK2* in PDCD, they provide a biological rationale for considering *DCLK2* as a potential modifier of neurological disease severity, particularly in a disorder characterized by neuronal vulnerability to impaired energy metabolism.

The marked clinical heterogeneity of PDCD suggests that additional genetic factors may influence disease expression, particularly in consanguineous families. Analysis of shared autozygous or identity-by-descent (IBD) regions can facilitate the identification of rare variants that may contribute to phenotypic severity beyond the primary disease-causing variant.

The present study aimed to characterize the clinical and molecular features of two affected siblings from a consanguineous family with PDCD, identify the underlying genetic cause, and investigate rare variants within shared IBD regions as potential contributors to the observed phenotype. In particular, we identified a homozygous *DCLK2* variant within the shared IBD interval and evaluated its potential contribution to the severe neurological and visual manifestations observed in the family.

## 2. Methods

### 2.1. Human Subjects and Clinical Characterisation

Ethical approval for the study was obtained from the Kuwait Ministry of Health Research Ethics Committee (Ethics ID: 62/2013). The study was conducted with the participation of members of the affected family, and written informed consent was obtained from the adult participants for the review of medical records and genetic investigations. Blood samples were collected from the two affected individuals and their unaffected parents.

The affected siblings and their family members were initially evaluated at Al-Jahra Hospital by pediatricians based on their clinical presentation and relevant medical history. The family was subsequently referred to the Human Genetics Unit, Faculty of Medicine, Kuwait University, for specialist clinical and genetic evaluation and diagnostic confirmation. The affected siblings underwent detailed clinical assessment and molecular investigation, and the diagnosis was established through integration of the clinical, biochemical, radiological, and genetic findings.

Clinical information was obtained through direct clinical assessment and review of medical records, hospital admissions, and outpatient follow-up records. Clinical, biochemical, neurological, ophthalmological, and radiological findings were systematically reviewed to document the age at onset, longitudinal progression, and severity of manifestations in the two affected siblings. Relevant laboratory results, microbiological findings, ventilatory requirements, major interventions, and available brain neuroimaging findings were compiled from the medical records. The clinical and laboratory domains assessed are summarized in Table 1, while the principal demographic and birth characteristics of the affected siblings are presented in Table 2.

### 2.2. Genomic DNA Extraction and Exome Sequencing

Genomic DNA was extracted from peripheral whole blood using the QIAamp DNA Mini Kit (Qiagen, Hilden, Germany). DNA concentration was determined by ultraviolet spectrophotometry using a NanoDrop ND-1000 spectrophotometer (Thermo Fisher Scientific/NanoDrop Technologies, Wilmington, DE, USA).

**Figure 1:**
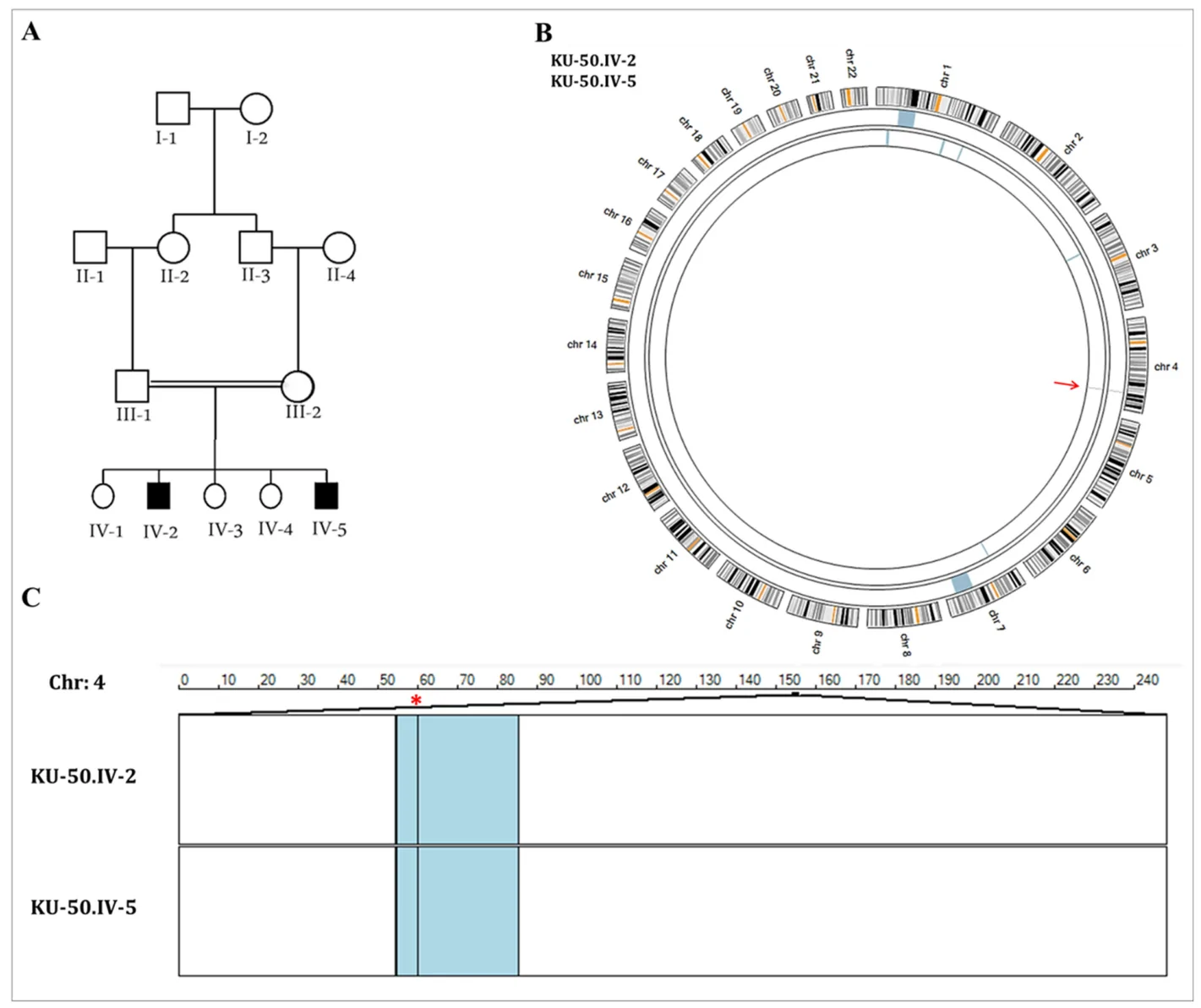
Genetic linkage and autozygosity analysis in Family KU-50 (**A**) Pedigree of Family KU-50 showing the two affected siblings with pyruvate dehydrogenase complex deficiency (PDCD). (**B**) Agile Multi-Ideogram plots for individuals KU-50.IV-2 and KU-50.IV-5. In individual KU-50.IV-2, two regions of runs of homozygosity (ROHs) are observed on chromosomes 1 and 7. Both affected individuals share a discrete, narrowly defined region of autozygosity on chromosome 4 (black line), indicated by the red arrow. (**C**) Multiple linkage analyses of chromosome 4 for the two affected siblings were performed. The shared identical-by-descent (IBD) interval on chromosome 4 was estimated to span chr4:148,334,756–154,560,476 (~6 Mb). The physical location of *DCLK2* (chr4:150,078,445–150,257,438), which lies within the shared IBD interval, is highlighted by a red asterisk. Each row represents the genotypes across chromosome 4 for one affected individual. Concordant autozygous alleles are shown in blue, while rare concordant autozygous alleles are shown in black.

Whole-exome sequencing (WES) was performed for the two affected male siblings, KU-50.IV-2 and KU-50.IV-5 (Figure 1A). Exonic regions were enriched using SureSelect Human All Exon v6 hybridization-capture reagents (Agilent Technologies, Santa Clara, CA, USA), according to the manufacturer’s protocol. The enriched libraries were sequenced on a HiSeq 2500 platform (Illumina, San Diego, CA, USA). Variant call format (VCF) files generated from the exome sequencing pipeline were subsequently used for downstream variant screening and linkage/autozygosity analyses.

### 2.3. Autozygosity Mapping and Variant Screening

Autozygosity mapping was performed using the exome sequencing data, as previously described [35]. Homozygous regions shared between the affected siblings were identified to assess genomic regions that could contain candidate variants associated with the phenotype. AgileMultiIdeogram software (version 3) was used to visualize the identified homozygous intervals across the autosomal chromosomes and to facilitate assessment of shared regions between the affected individuals [36]. The analysis was based on the human genome reference assembly GRCh38 (UCSC Genome Browser).

The identified shared homozygous intervals were subsequently examined for candidate variants. Particular attention was given to variants located within regions shared by both affected siblings and compatible with the inheritance pattern observed in the family. The chromosome 4 identical-by-descent (IBD) interval identified through this analysis was subsequently evaluated by multipoint linkage analysis using the AgileVCFMapper software (version 3).

### 2.4. Variant Confirmation and Segregation Analysis

Candidate variants identified by exome sequencing were confirmed by Sanger sequencing, and segregation analysis was performed in the two affected siblings (KU-50.IV-2 and KU-50.IV-5) and their parents (KU-50.III-1 and KU-50.III-2). Primers were designed using Primer3 software (https://primer3.org/; accessed 1 October 2025). Exonic sequences used for primer design were obtained from the UCSC Genome Browser (http://genome.ucsc.edu/; accessed 1 November 2025). For the *PDHA1* variant in exon 6 (c.754C>G; p.Leu252Val; rs1555934383), the forward primer was 5′-ACCCAAAGCTCGGTTTTAGA-3′ and the reverse primer was 5′-CCATTCCATCCACCTGGTAA-3′. The *DCLK2* variant identified in exon 1 (c.101G>A; p.Ser34Asn) was similarly confirmed by Sanger sequencing. The forward primer was 5′-TGCGGACACTTTTAGCTGAG-3′, and the reverse primer was 5′-CGGGTGAGCTCTATGAGGAG-3′. Sequencing reactions and subsequent analysis were performed as previously described [35,37]. The identified variants were evaluated in the available family members to determine their segregation with the disease phenotype and to establish their respective inheritance patterns.

### 2.5. In Silico Characterization of the PDHA1 and DCLK2 Variants

In silico analyses were performed to assess the predicted functional and pathogenic effects of the identified *PDHA1* and *DCLK2* missense variants. Prediction scores were obtained from dbNSFP version 4.1a [38], which provides aggregated annotations from multiple computational tools for nonsynonymous single-nucleotide variants.

The *PDHA1* c.754C>G (p.Leu252Val) variant was evaluated using M-CAP, FATHMM, MetaLR, MutationTaster, MutPred, and REVEL. M-CAP was used to estimate the likelihood that the missense variant is pathogenic [39], whereas FATHMM incorporates sequence conservation and hidden Markov models to predict the functional consequences of amino acid substitutions [40]. MetaLR integrates multiple annotation features using a logistic regression framework [41]. MutationTaster incorporates information relating to evolutionary conservation, protein features, and potential effects on splicing and other molecular processes [42]. MutPred was used to estimate the probability of pathogenicity and potential molecular consequences of the amino acid substitution [43]. REVEL, an ensemble predictor developed for the assessment of rare missense variants, was also included [44].

The *DCLK2* c.101G>A (p.Ser34Asn) variant was evaluated using GenoCanyon, the likelihood ratio test (LRT), PolyPhen-2 (HDIV model), MetaSVM, and FATHMM-MKL (coding) [45,46,47,48]. GenoCanyon was used to assess the functional potential of genomic positions based on evolutionary conservation and genomic features [45]. LRT evaluates evolutionary constraint by comparing conserved and neutral models [46]. PolyPhen-2 was used to predict the potential structural and functional impact of the amino acid substitution [47]. MetaSVM and FATHMM-MKL integrate multiple sequence- and genomic-level features to estimate the likelihood of deleterious effects [48].

### 2.6. Brain MRI Protocol

Brain and orbital magnetic resonance imaging (MRI) was performed without intravenous contrast at 18 years of age at Al-Jahra Hospital, Kuwait. MRI was acquired on a 1.5-Tesla system (GE Healthcare, Chicago, IL, USA). MRI was acquired on a 1.5-Tesla system (GE Healthcare, USA). The imaging protocol included axial T1-weighted, T2-weighted, T2-weighted fluid-attenuated inversion recovery (FLAIR) with fat suppression, T1-weighted and T2-weighted fat-suppressed sequences, diffusion-weighted imaging (DWI) with apparent diffusion coefficient (ADC) mapping, and susceptibility-weighted angiography (SWAN). Coronal and sagittal T2-weighted and T2-weighted fat-suppressed sequences were also obtained, together with coronal short-tau inversion recovery (STIR) and sagittal oblique T2-weighted fat-suppressed sequences targeted to the orbits. Three-dimensional axial time-of-flight (TOF) spoiled gradient-echo (SPGR) fat-suppressed magnetic resonance angiography (MRA) was additionally performed.

## 3. Results

### 3.1. Clinical Characteristics of the Affected Siblings

The study included two affected male siblings from the KU-50 family, KU-50.IV-2 and KU-50.IV-5, born to consanguineous parents of Bedouin ancestry from the Arabian Peninsula. Their clinical presentation was consistent with primary pyruvate dehydrogenase complex deficiency (PDCD), characterized by early-onset metabolic disturbances, neurological involvement, developmental impairment, and recurrent episodes of clinical deterioration. Their demographic and birth characteristics are summarized in Table 2.

### 3.2. Neurological and Metabolic Course

KU-50.IV-2 presented with infantile generalized tonic–clonic seizures, which subsequently resolved after approximately one year, followed by recurrent episodes of ataxia and dysarthria, particularly during intercurrent illness. KU-50.IV-5 had no reported seizures but developed recurrent episodes of hypotonia, tremors, dysarthria, and abnormal eye movements from early childhood. Both siblings experienced recurrent neurological or metabolic decompensations, generally associated with intercurrent illness. The detailed neurological and early metabolic course is presented in Table 3.

Both siblings also developed persistent metabolic abnormalities and significant systemic complications (Table 4). Chronic or intermittent lactic acidosis was documented in both patients, with recurrent episodes of metabolic decompensation. The systemic complications differed between the siblings: KU-50.IV-2 developed severe obesity, type 2 diabetes mellitus, hypertension, obstructive sleep apnea, and sensorineural hearing loss, whereas KU-50.IV-5 had a predominantly respiratory course characterized by recurrent respiratory failure and prolonged ventilatory dependence. The medical records confirmed that muscle biopsy and fibroblast PDH enzyme studies had been performed for patient KU-50.IV-2 overseas, with the reported findings consistent with PDH deficiency. The chronological clinical course and major disease milestones of both affected siblings are provided in the Supplementary Section, Table S1A,B, including the timing of neurological, metabolic, respiratory, and systemic complications.

### 3.3. Genetic Findings and Family Pedigree

This study investigated a consanguineous family from the Arabian Peninsula. The KU-50 family comprised two affected male siblings (KU-50.IV-2, aged 18, and KU-50.IV-5, aged seven) and three unaffected siblings (see Figure 1A). The affected individuals were born to first-cousin parents, which is consistent with the consanguineous pedigree. Genetic analysis revealed a hemizygous *PDHA1* missense variant (c.754C>G; p.Leu252Val) in both affected males, which is consistent with X-linked *PDHA1*-related pyruvate dehydrogenase complex deficiency (PDCD). Segregation analysis also supported the observed inheritance pattern. Genome-wide autozygosity analysis was then performed to identify regions of shared homozygosity between the affected siblings. This analysis identified a shared region on chromosome 4, which was then evaluated using linkage and variant analyses (Figure 1B,C). The detailed characterization of the shared homozygous region and the *DCLK2* variant identified within this interval is presented in Section 3.8, Section 3.9 and Section 3.10, respectively.

### 3.4. Hospital Course and Outcome

Both siblings required repeated hospital admissions because of metabolic decompensation, respiratory deterioration, and infections. KU-50.IV-2 experienced multiple respiratory and metabolic crises requiring PICU care and intermittent non-invasive or invasive ventilatory support. His treatment included sodium bicarbonate, mitochondrial-directed therapy, BiPAP support, and antiepileptic medication (Table 5). The chronological clinical course and major disease milestones of KU-50.IV-2 and KU-50.IV-5 are summarized in Supplementary Table S1A and S1B, respectively.

KU-50.IV-5 had a more severe respiratory course, with recurrent infections, hypercapnic respiratory failure, prolonged invasive ventilation, and tracheostomy. Infectious complications included MRSA and *Pseudomonas aeruginosa*, with repeated positive cultures associated with the tracheostomy. He subsequently developed tracheostomy obstruction with granuloma formation and died at 7 years of age from acute respiratory failure secondary to tracheostomy obstruction (Table 5). Additional laboratory, neuroimaging, neurological, and ophthalmological findings are provided in Supplementary Table S2A–C, including detailed investigation results for both affected siblings. A detailed summary of hospitalizations, respiratory complications, infections, intensive care interventions, and treatment received by both siblings is provided in Supplementary Table S3.

### 3.5. Neuroimaging Findings

**Figure 2:**
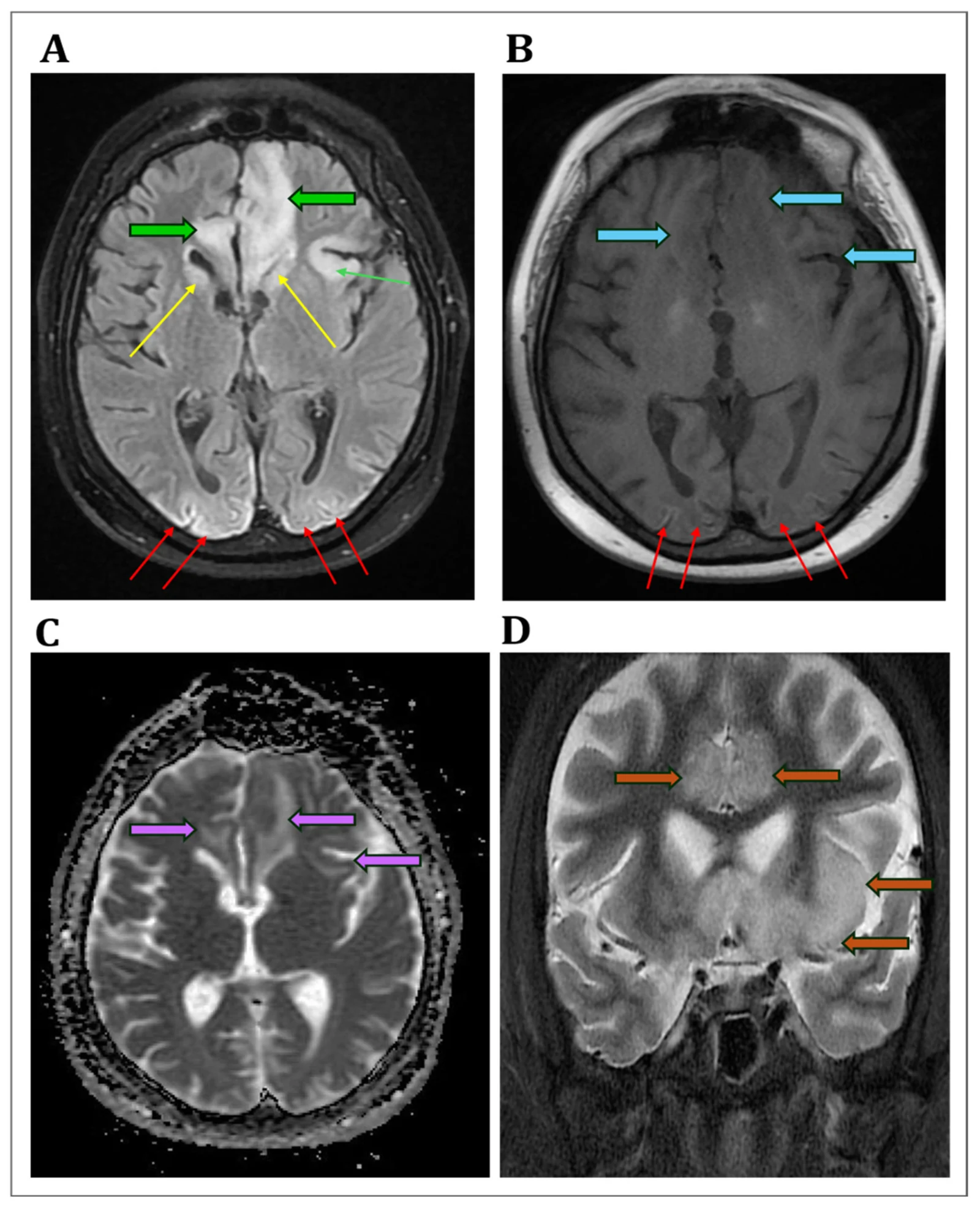
Brain MRI findings in KU-50.IV-2 demonstrating cortical and subcortical involvement suggestive of metabolic encephalopathy. (**A**): Axial fluid-attenuated inversion recovery (FLAIR) image, serving as the primary lesion-defining sequence, demonstrating bilateral cortical and subcortical signal abnormalities involving the frontal lobes with left-sided predominance (green arrows), bilateral heads of the caudate nuclei (yellow arrows), and the left insular cortex (light green thin arrow). Abnormal signal intensities are also seen involving the visual cortex (parieto-occipital areas) (red arrows) in the form of high T2WI/FLAIR signal intensity with corresponding slightly high T1 signal (red arrows in (**A**,**B**)) without diffusion restriction, signifying possible chronic ischemic changes with cortical laminar necrosis. (**B**): Axial T1-weighted image showing corresponding low T1 signal intensity within the affected regions, supporting a chronic and metabolic etiology (light blue arrows). (**C**): Apparent diffusion coefficient (ADC) map demonstrating absence of diffusion restriction, arguing against acute ischemic changes and favoring a metabolic or mitochondrial chronic process. (Purple arrows) (**D**): Coronal short tau inversion recovery (STIR) image illustrating the anatomical extent and relative asymmetry of the lesions (orange arrows), complementing the axial FLAIR findings. Remark: Figures obtained from a non-enhanced MRI of the brain and orbits. Motion artifact degraded some of the available images.

**Figure 3:**
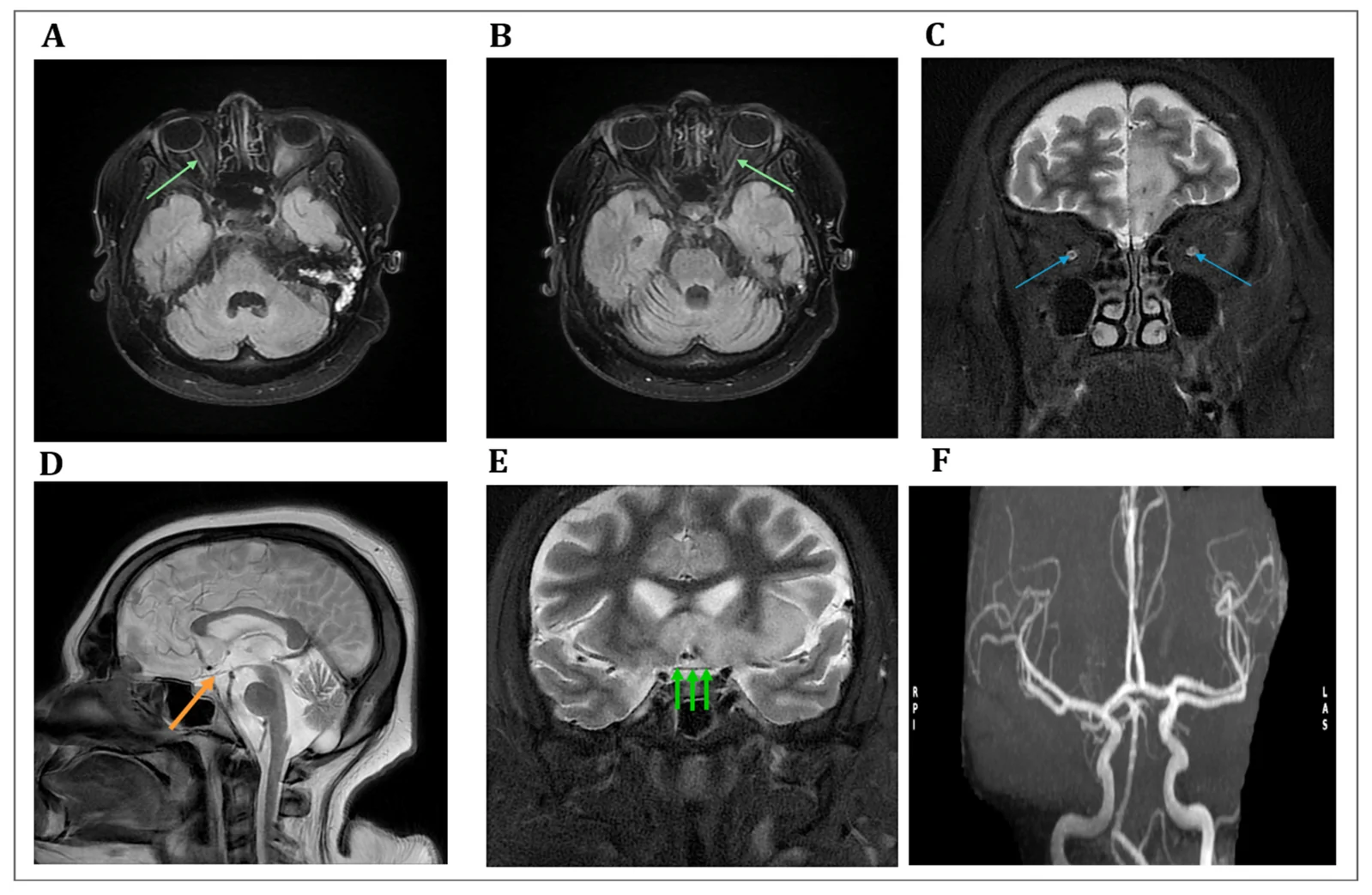
Brain MRI findings in KU-50.IV-2 demonstrating overall gross reduction in the volume of both optic nerves: (**A**,**B**): Axial T2-weighted fluid-attenuated inversion recovery (FLAIR) fat-suppressed images demonstrate a gross reduction in the volume of both optic nerves (green arrows). (**C**): Coronal short tau inversion recovery (STIR) image demonstrates an overall gross reduction in the volume of both optic nerves while preserving normal intrinsic signal intensity (light blue arrows). (**D**): Sagittal T2-weighted image demonstrating a gross reduction in the volume of the optic chiasm (orange arrow). (**E**): Coronal short tau inversion recovery (STIR) image further demonstrates the gross reduction in the volume of the optic chiasm (green arrows). (**F**): The intracranial internal carotid and vertebral arteries are normal in caliber and course. The circle of Willis is intact. No MR evidence of significant intracranial arterial stenosis, aneurysm, or arteriovenous malformation is identified.

Neuroimaging demonstrated central nervous system abnormalities in KU-50.IV-2. Brain MRI showed basal ganglia signal abnormalities compatible with Leigh changes, together with multifocal cortical and subcortical abnormalities involving the bilateral anterior frontal lobes, particularly the parafalcine regions, caudate heads, left insular cortex, and bilateral parieto-occipital/visual cortices (Figure 2). The affected regions showed high signal intensity on FLAIR and STIR sequences and corresponding low signal intensity on T1-weighted images. No diffusion restriction was observed on ADC maps, and susceptibility-weighted imaging showed no evidence of hemorrhage, microbleeding, or calcification (Figure S1). The posterior cortical abnormalities demonstrated chronic signal changes, including features suggestive of cortical laminar necrosis. Marked reduction in the volume of both optic nerves and the optic chiasma was also noted, with preserved intrinsic signal intensity (Figure 3). The imaging findings are summarized in Table 6.

### 3.6. Ophthalmological Findings

An ophthalmological assessment of KU-50.IV-2 revealed severe bilateral visual impairment. Un-aided visual acuity was counting fingers at a distance of 50 cm in both eyes. There was poor fixation and a large-angle left exotropia of approximately 50 prism diopters. The anterior segments were normal, as were the ocular media. Fundoscopy revealed bilateral optic disc pallor, which was consistent with optic atrophy. The macula appeared clinically normal.

**Figure 4:**
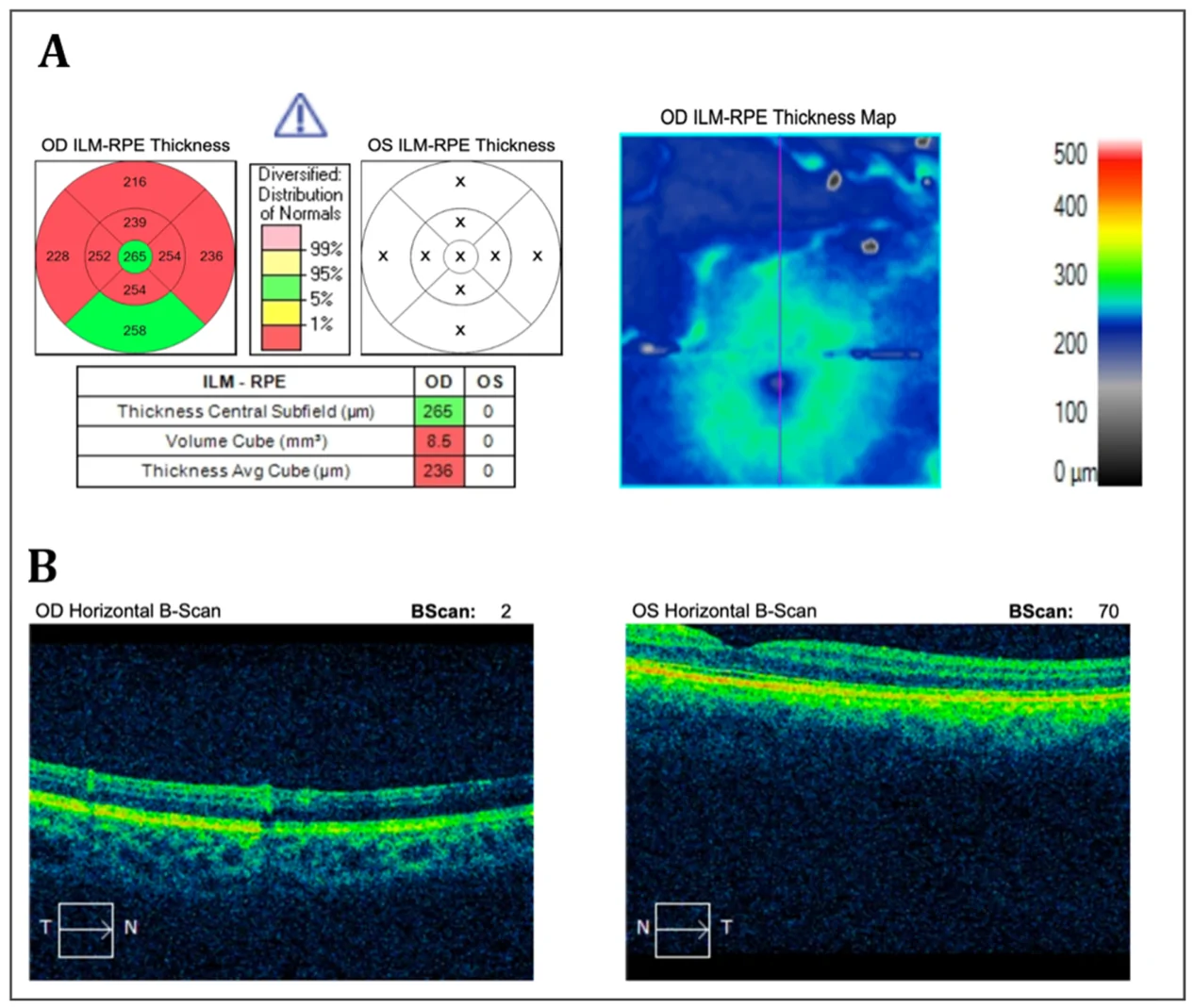
Macular spectral-domain optical coherence tomography (OCT) in KU-50.IV-2: (**A**) ILM–RPE thickness analysis. In the right eye (OD), the central subfield thickness (265 µm) is within normal limits. However, most inner and outer sectors fall below the first percentile of the normative database (red), indicating reduced cube volume and average cube thickness. Thickness could not be computed for the left eye (OS) due to poor fixation (sectors marked ‘X’). (**B**) Horizontal B-scans of the right and left eyes, acquired at different macular levels (OD: B-scan 2 of 128; OS: B-scan 70 of 128), are shown for qualitative assessment only. The foveal depression is only captured in the OS scan. Image acquisition was limited by poor fixation and large-angle exotropia (signal strength 6/10; automated foveal centration not achieved).

Spectral-domain macular optical coherence tomography (OCT) was limited by poor fixation and large-angle exotropia, as well as limited cooperation (signal strength 6/10 and automated foveal centration not achieved). In the right eye, central subfield thickness was within normal limits (265 µm); however, most of the parafoveal and perifoveal sectors fell below the first percentile of the normative database. This was indicative of reduced cube volume (8.5 mm^3^) and average cube thickness (236 µm), indicating thinning of the macula in the para- and perifoveal regions. Thickness analysis could not be computed for the left eye. On qualitative review, the horizontal B-scan of the left eye through the fovea showed a preserved foveal contour without obvious thinning (Figure 4). Optic nerve head OCT was not available.

Neuroimaging revealed involvement of both the anterior and posterior visual pathways. Non-contrast MRI of the brain and orbits showed a reduced volume in both the optic nerves and the optic chiasm, with a preserved intrinsic signal intensity. This is consistent with optic atrophy (see Figure 3). Additionally, cortical and subcortical signal abnormalities involving the parietal and occipital regions, without diffusion restriction, were suggestive of chronic cortical injury (Figure 2).

Taken together, the clinical, OCT and MRI findings were consistent with cerebral (cortical) visual impairment with concomitant bilateral optic atrophy, indicating combined involvement of the anterior and posterior visual pathways (see Table 7).

### 3.7. Identification of a Shared Homozygous Region and the DCLK2 Variant

Since both affected individuals were male and had a phenotype strongly suggestive of primary PDCD, whole-exome sequencing was performed in KU-50.IV-2 and KU-50.IV-5. Genome-wide autozygosity analysis identified several homozygous intervals. KU-50.IV-2 carried individual homozygous intervals on chromosomes 1 and 7.

Importantly, both affected siblings shared a subtle region of homozygosity on chromosome 4 that was barely discernible on the initial genome-wide analysis (Figure 1B). Subsequent multipoint linkage analysis resolved this region more clearly and defined a shared interval of approximately 6 Mb on chromosome 4 (chr4:148,334,756–154,560,476) (Figure 1C).

Interrogation of the shared interval using the exome data identified a homozygous missense variant in *DCLK2*, c.101G>A (p.Ser34Asn). The variant lies within the shared IBD region at chr4:150,078,445–150,257,438. It was not associated with an rs identifier in the NCBI dbSNP database at the time of analysis. The two affected siblings were homozygous for the variant.

### 3.8. Identification of the Hemizygous PDHA1 Variant

**Figure 5:**
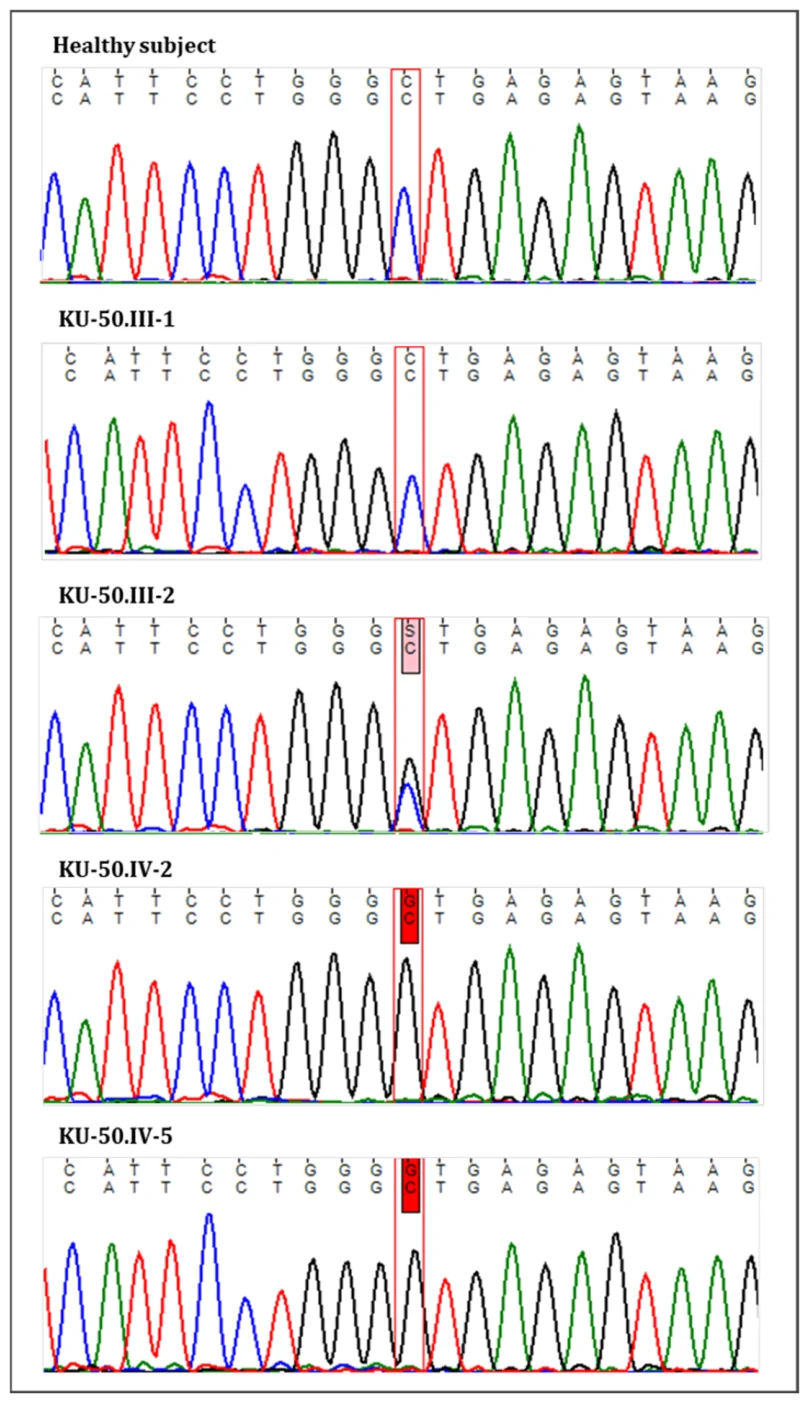
*PDHA1* variant chromatographs for the two affected siblings and the parents of Family KU-50. Chromatogram sequences for the healthy subject, the parents (KU-50.III-1 and KU-50.III-2), and the two PDCD patients (KU-50.IV-2 and KU-50.IV-5) are shown. The two affected siblings harbor a novel hemizygous missense variant c.754C>G (p.Leu252Val; rs1555934383). The father (KU-50.III-1) carries the wild-type PDHA1 allele, while the mother (KU-50.III-2) is a heterozygous carrier, consistent with X-linked segregation of a PDCD-causing variant. At the pathogenic variant locus, pink rectangles indicate heterozygous variants, whereas red rectangles indicate homozygous pathogenic variants. The two red lines indicate the nucleotide position that differs from the wild-type allele.

The clinical phenotype and the occurrence of disease in two male siblings prompted additional examination of the X chromosome. Screening of the exome data for hemizygous variants identified a missense variant in exon 6 of *PDHA1*, c.754C>G (p.Leu252Val; rs1555934383), located at chrX:19,343,893–19,361,707. The *PDHA1* variant is consistent with the established X-linked inheritance pattern of primary PDCD and provides a molecular explanation for the core disease phenotype. Sanger sequencing confirmed the presence of the variant in both affected siblings (Figure 5), who were hemizygous for the change. The identification of the *PDHA1* variant alongside the homozygous *DCLK2* variant provided the basis for examining whether the chromosome 4 variant could contribute to the clinical variability observed in the family.

### 3.9. Segregation Analysis of the Two Variants in PDHA1 and DCLK2

**Figure 6:**
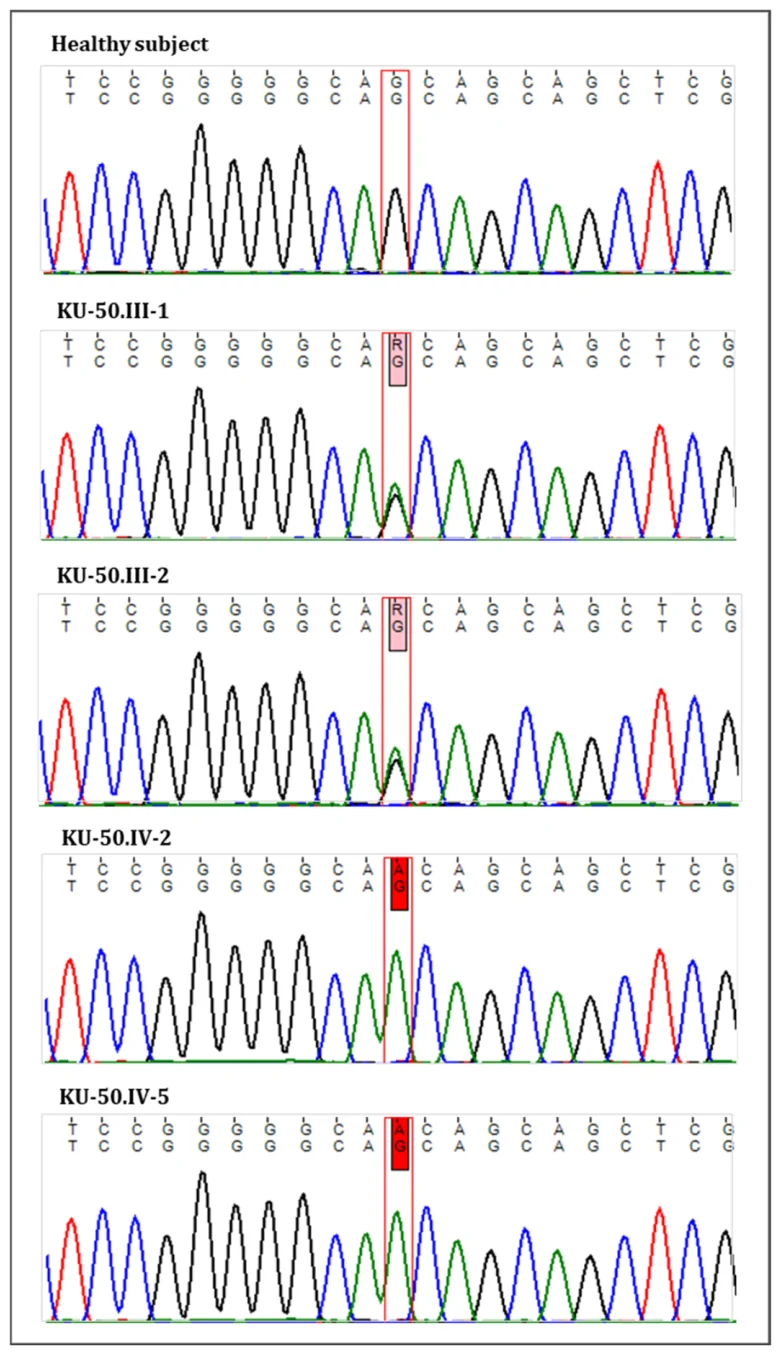
*DCLK2* variant chromatographs for the two affected siblings and the parents of Family KU-50. Chromatogram sequences for the healthy subject, the parents (KU-50.III-1 and KU-50.III-2), and the two PDCD patients (KU-50.IV-2 and KU-50.IV-5) are shown. The two affected siblings harbor a novel homozygous missense variant c.101G>A (p.Ser34Asn). The parents (KU-50.III-1 and KU-50.III-2) are heterozygous carriers of the variant, consistent with autosomal recessive inheritance. The homozygous *DCLK2* variant in the affected individuals provides strong genetic evidence for a “two-hit” model, in which *DCLK2* acts as a genetic modifier contributing to the phenotypic severity of PDCD. At the pathogenic variant locus, pink rectangles indicate heterozygous variants, whereas red rectangles indicate homozygous pathogenic variants. The two red lines indicate the nucleotide position that differs from the wild-type allele.

Segregation analysis confirmed that both variants were present in the family in patterns consistent with their respective modes of inheritance (Figure 5 and Figure 6). The two affected siblings carried the hemizygous *PDHA1* (c.754C>G; p.Leu252Val) variant (Figure 5) and were homozygous for the *DCLK2* (c.101G>A; p.Ser34Asn) variant (Figure 6). The mother, KU-50.III-2, was heterozygous for both variants. The father, KU-50.III-1, carried the wild-type *PDHA1* allele and was heterozygous for the *DCLK2* variant. The segregation pattern therefore supports maternal transmission of the X-linked *PDHA1* variant and autosomal recessive inheritance of the *DCLK2* variant from both parents. These findings establish the co-occurrence of the two variants in the affected siblings. The *PDHA1* variant provides the primary molecular explanation for the X-linked PDCD phenotype, whereas the homozygous *DCLK2* variant represents an additional inherited genetic finding located within the shared IBD interval.

### 3.10. In Silico Characterisation of the PDHA1 and DCLK2 Variants

Multiple computational predictors were used to assess the potential functional effects of the *PDHA1* c.754C>G (p.Leu252Val) and *DCLK2* c.101G>A (p.Ser34Asn) variants. For *PDHA1*, most predictors supported a deleterious effect, including FATHMM, MutationTaster, MetaLR, and MutPred, whereas M-CAP and REVEL provided weaker or borderline predictions. For *DCLK2*, GenoCanyon, the likelihood ratio test, PolyPhen-2, and FATHMM-MKL supported a potential damaging effect, while MetaSVM predicted a more tolerant effect. Overall, the computational findings provided supportive evidence for a potential functional effect of both variants. Detailed prediction scores and interpretations are provided in Supplementary Tables S4 and S5.

### 3.11. ACMG/AMP Classification of the PDHA1 and DCLK2 Variants

The identified *PDHA1* and *DCLK2* variants were evaluated according to the American College of Medical Genetics and Genomics/Association for Molecular Pathology (ACMG/AMP) sequence-variant interpretation framework. The assessment incorporated the available population, computational, segregation, phenotype, and functional evidence. The evidence supporting the classification of each variant is summarized in Table 8 and Table 9.

#### 3.11.1. Classification of the PDHA1 Variant

The *PDHA1* c.754C>G (p.Leu252Val) variant was identified in a hemizygous state in both affected male siblings and was absent from the unaffected father. The variant segregated with the disease phenotype through the mother, who was heterozygous for the variant. Multiple computational predictors supported a deleterious effect, although predictions were not completely concordant. In addition, the clinical phenotype of the two affected individuals was highly consistent with *PDHA1*-associated primary PDCD.

Taken together, the available genetic, clinical, computational and tissue-based evidence supports the pathogenic interpretation of the *PDHA1* c.754C>G (p.Leu252Val) variant. The individual ACMG/AMP evidence criteria applied to this variant are presented in Table 8.

#### 3.11.2. Classification of the *DCLK2* Variant

The *DCLK2* c.101G>A (p.Ser34Asn) variant was identified in a homozygous state in both affected siblings and in a heterozygous state in each parent. The variant lies within the shared chromosome 4 identical-by-descent interval identified by genome-wide autozygosity and subsequent linkage analysis. Several computational tools predicted a potentially deleterious effect, although the results were heterogeneous, with some predictors supporting a damaging effect and others indicating tolerance.

The *DCLK2* variant has not previously been established as a cause of PDCD, and no functional assay was performed to directly demonstrate an effect of the p.Ser34Asn substitution on DCLK2 protein function. Therefore, despite its homozygous segregation, localization within the shared IBD interval, and supportive computational evidence, the available data are insufficient to establish pathogenicity under the ACMG/AMP framework. The variant is consequently best reported as a variant of uncertain significance, while its potential contribution to disease severity is considered as a hypothesis requiring functional validation.

**Figure 7:**
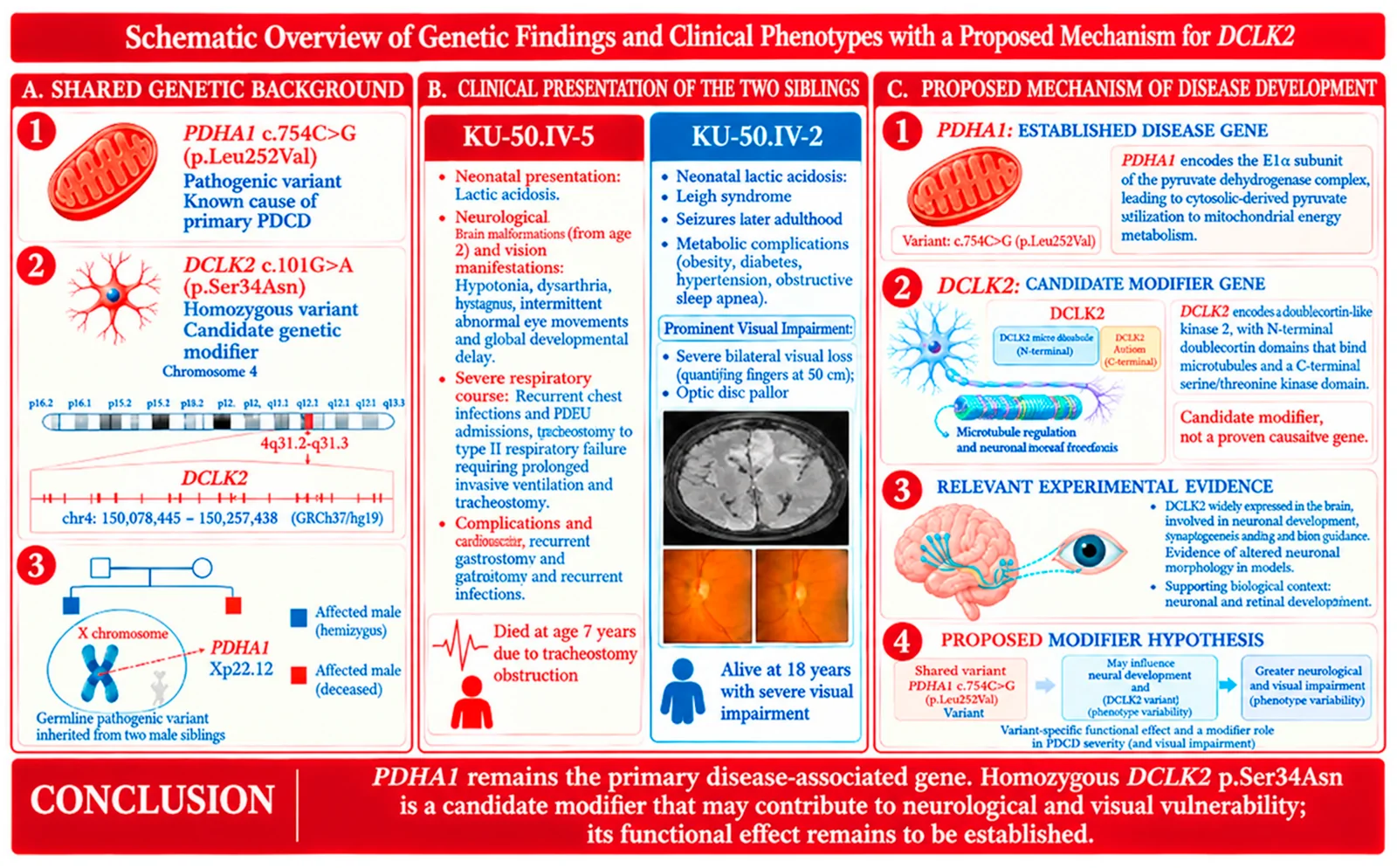
Proposed two-hit model involving *PDHA1* and *DCLK2*. (**A**) Inheritance of the germline pathogenic *PDHA1* variant and the homozygous *DCLK2* variant in the two affected male siblings, with the *DCLK2* variant consistent with a founder effect in the family. (**B**) Clinical presentation of the two affected siblings. (**C**) Interpretation of the genetic and clinical findings, supporting a potential modifier role of the *DCLK2* variant in the phenotypic severity of *PDHA1*-associated primary pyruvate dehydrogenase complex deficiency. Abbreviations: DCLK2, doublecortin-like kinase 2; PDCD, pyruvate dehydrogenase complex deficiency; PDHA1, pyruvate dehydrogenase E1 alpha 1 subunit. EHF, ETS homologous factor; MRI, magnetic resonance imaging.

The ACMG/AMP assessment therefore supports a substantially stronger level of evidence for the *PDHA1* variant than for the *DCLK2* variant. The *PDHA1* variant provides the established molecular basis for the primary PDCD phenotype, whereas the *DCLK2* variant represents an additional homozygous genetic finding that may contribute to phenotypic severity. The latter interpretation remains provisional in the absence of direct functional evidence. The genetic findings, clinical presentation of the two affected siblings, and the proposed interpretation of the combined *PDHA1* and *DCLK2* variants are summarized in Figure 7.

## 4. Discussion

Primary pyruvate dehydrogenase complex deficiency (PDCD) is a genetically heterogeneous mitochondrial disorder with a broad clinical spectrum, ranging from neonatal lactic acidosis and Leigh syndrome to later-onset neurological, neuromuscular, and multisystem manifestations. Pathogenic variants have been identified in several genes encoding structural or regulatory components of the pyruvate dehydrogenase complex, including *PDHA1*, *PDHB*, *PDHX*, *DLAT*, *DLD*, *PDP1*, and *PDK3*, with *PDHA1* accounting for approximately 76–85% of reported cases. In the present family, both affected male siblings carried the same hemizygous *PDHA1* missense variant (c.754C>G; p.Leu252Val), inherited from their heterozygous mother, consistent with the established X-linked pattern of *PDHA1*-associated PDCD [1,49,50]. The clinical phenotype of both individuals, including neonatal lactic acidosis, neurological involvement, and Leigh brain abnormalities, was also consistent with *PDHA1*-related PDCD.

The genetic investigation was particularly informative because the family structure and clinical presentation initially pointed towards a straightforward X-linked disorder. Both affected individuals were male siblings, and the identification of a hemizygous *PDHA1* variant provided a convincing explanation for the primary disease. However, genome-wide autozygosity mapping revealed an additional shared homozygous region on chromosome 4. Autozygosity mapping is a well-established approach for identifying shared identical-by-descent (IBD) regions in consanguineous families and has been successfully used to identify autosomal recessive disease loci [35,51,52,53,54]. In this family, the shared chromosome 4 interval prompted further examination of the exome data and led to the identification of a homozygous *DCLK2* missense variant. This finding illustrates the value of considering additional genomic variation when the clinical course within a consanguineous family is more severe or variable than might be expected from a single established disease-causing variant.

Although the two affected brothers carried the same *PDHA1* and *DCLK2* variants, a direct comparison of their clinical phenotypes is limited by their different ages and disease courses. This is particularly true given that the younger brother died at the age of seven. The older sibling, KU-50.IV-2, experienced recurrent lactic acidosis and neurological episodes, including ataxia and dysarthria, together with basal ganglia abnormalities on neuroimaging characteristic of Leigh syndrome. He survived into late adolescence but developed significant multisystem complications, including morbid obesity, obstructive sleep apnea, hypertension, diabetes mellitus, respiratory insufficiency, and sensorineural hearing loss. In contrast, KU-50.IV-5 developed severe neurological and respiratory disease at a younger age, with hypotonia, tremors, dysarthria, recurrent encephalopathic episodes, progressive respiratory failure, and prolonged ventilatory dependence. His clinical course ultimately resulted in death following complications associated with tracheostomy. The differences in age at onset, neurological progression, respiratory involvement and overall outcome emphasize the variation in clinical presentation and disease progression among individuals with closely related *PDHA1* defects.

The neuroimaging findings in KU-50.IV-2 demonstrated basal ganglia abnormalities with multifocal cortical and subcortical involvement extending to the bilateral frontal, insular, and parieto-occipital regions. The relatively symmetric distribution and absence of diffusion restriction are compatible with a chronic metabolic or mitochondrial encephalopathic process, with superimposed chronic ischemic-type changes.

The visual phenotype of KU-50.IV-2 was severe, affecting both the anterior and posterior visual pathways. Bilateral optic disc pallor, together with reduced optic nerve and chiasmal volume on MRI scans, indicates established optic atrophy. Meanwhile, bilateral parietal-occipital signal abnormalities suggest a concomitant cerebral (cortical) component. Optic atrophy has been reported in PDCD [14], which is consistent with the recognised vulnerability of retinal ganglion cells and their axons to impaired mitochondrial energy metabolism [55,56,57,58].

However, combined anterior and posterior visual pathway involvement of this severity is uncommon. It is not possible to determine from the available data whether the optic atrophy represents a primary mitochondrial optic neuropathy, retrograde transsynaptic degeneration secondary to cortical injury, or both. The large-angle left exotropia is most likely sensory, reflecting profound early visual loss and impaired binocular fusion. A contribution from mitochondrial extraocular myopathy cannot be excluded, although restriction of ocular motility was not documented. The severity and extent of visual pathway involvement may suggest the influence of additional genetic factors on the expression of this phenotype. In this context, the homozygous *DCLK2* p.Ser34Asn variant may contribute to the neurological and visual phenotype, consistent with a potential modifier role.

Both affected siblings were homozygous for the novel *DCLK2* missense variant c.101G>A (p.Ser34Asn), which lies within the shared chromosome 4 IBD interval. The variant was inherited from heterozygous parents and was present in both affected individuals, supporting its segregation within the family. Although the available evidence is insufficient to establish *DCLK2* c.101G>A (p.Ser34Asn) as a primary disease-causing variant, its localization within the shared IBD region, homozygous state in both affected siblings, and potential biological relevance make it a plausible candidate modifier of the *PDHA1*-associated phenotype. In an individual already affected by mitochondrial energy failure, increased susceptibility to stress-induced neuronal injury could potentially contribute to a more severe neurological phenotype.

The biological functions of DCLK2 provide a compelling rationale for investigating its potential contribution to the neurological phenotype observed in this family. DCLK2 is a neuronal microtubule-associated protein that contains N-terminal doublecortin domains, which are involved in microtubule binding, as well as a C-terminal serine/threonine kinase domain. This links cytoskeletal organization to intracellular signaling. Experimental studies have implicated DCLK2 and related doublecortin-like kinases in dendritic development, axonal growth, neuronal survival, and regenerative responses following neuronal injury. Studies of retinal ganglion cells, in particular, have demonstrated that DCLK2 overexpression promotes growth cone reformation and axonal regeneration following optic nerve injury, thereby highlighting its importance for neuronal integrity and visual pathways [27,59].

The potential modifying role of *DCLK2* is biologically plausible given its involvement in neuronal stress responses. Experimental studies of chronic cerebral ischemia and oxygen-glucose deprivation have shown that altered *DCLK2* expression influences neuronal injury and survival [25,26,27]. This is particularly relevant in PDHA1 deficiency, where impaired energy metabolism places neurons under metabolic stress.

DCLK2 has also been implicated in stress-associated inflammatory and cell-death pathways through EHF-dependent signaling and caspase-associated neuronal pyroptosis [28,29,30], with the CPEB4-DCLK2-EHF pathway further linked to neuronal injury responses [31,32,33,34]. Although these findings do not establish a direct interaction between DCLK2 and the pyruvate dehydrogenase complex, they provide a plausible framework whereby altered DCLK2 function could modify neuronal vulnerability to metabolic stress. This may be particularly relevant to the severe neurological and visual phenotype observed in the present family.

In the present family, the coexistence of the homozygous DCLK2 p.Ser34Asn variant and an established, disease-associated PDHA1 variant suggests that altered DCLK2-dependent neuronal processes may modify the neurological and visual manifestations of primary pyruvate dehydrogenase complex deficiency.

On this basis, we propose that the homozygous DCLK2 p.Ser34Asn variant may act as a genetic modifier rather than as the primary cause of PDCD. The hemizygous *PDHA1* variant establishes the underlying X-linked metabolic disorder, while the additional biallelic *DCLK2* variant may contribute to the severity or spectrum of neurological and visual manifestations. Therefore, we propose *DCLK2* as a candidate modifier gene rather than an independent cause of the primary disorder. This hypothesis must be tested through variant-specific functional studies examining kinase activity, microtubule interactions, neuronal stress responses, and relevant visual system functions.

The findings also illustrate the value of comprehensive genomic analysis in consanguineous families, where multilocus variation may coexist with the primary disease-causing variant. In the present family, the combination of exome sequencing, autozygosity mapping, and segregation analysis identified both the hemizygous *PDHA1* variant and the homozygous *DCLK2* variant within a shared IBD interval. This approach may be particularly informative when the clinical phenotype is unusually severe or incompletely explained by a single pathogenic variant.

Taken together, the findings support a proposed two-hit model in which the primary *PDHA1* defect is accompanied by the homozygous *DCLK2* p.Ser34Asn variant, which may contribute to phenotypic severity. Functional studies will be required to determine whether and how this *DCLK2* variant modifies neuronal responses to impaired energy metabolism.

## Figures and Tables

**Table 1:** Clinical, laboratory, and molecular domains assessed in the study.

Domain	Parameters Assessed
Demographics and birth history	Gestational age, birth weight, mode of delivery, neonatal complications, and Apgar scores
Neurological and developmental features	Seizure history, ataxia, dysarthria, hypotonia, eye movement abnormalities, brain MRI findings, and brain CT findings
Metabolic and systemic features	Lactic acidosis, metabolic crises, obesity, diabetes mellitus, hypertension, sleep apnoea, and hearing impairment
Hospital admissions and interventions	Pediatric intensive care unit (PICU) admissions, invasive and non-invasive mechanical ventilation, tracheostomy, major infections, medications, and surgical procedures
Laboratory investigations	Arterial blood gas (ABG) analyses, complete blood counts (CBC), coagulation profiles, liver function tests, renal function tests, and microbiological cultures
Genetic and molecular investigations	*PDHA1* genetic analysis, identification and characterization of the *DCLK2* variant.

Abbreviations: ABG, arterial blood gas; CBC, complete blood count; CT, computed tomography; *DCLK2*, doublecortin-like kinase 2; MRI, magnetic resonance imaging; *PDHA1*, pyruvate dehydrogenase E1 alpha 1 subunit; PICU, paediatric intensive care unit.

**Table 2:** Demographic and birth characteristics of the two affected siblings.

Feature	KU-50.IV-2	KU-50.IV-5
Gender	Male	Male
Birth gestation	33 weeks	Term
Birth weight	2 kg	2.6 kg
Mode of delivery	LSCS (previous CS and placenta previa)	Vaginal, uncomplicated
Neonatal complications	Grade II respiratory distress syndrome, bilateral pneumothorax; NICU admission for 1 week	Respiratory distress syndrome; NICU admission
Apgar score	Good	Not specified

Abbreviations: CS, caesarean section; LSCS, lower-segment caesarean section; NICU, neonatal intensive care unit.

**Table 3:** Neurological manifestations and metabolic decompensation.

Feature	KU-50.IV-2	KU-50.IV-5
Seizures	Infantile generalized tonic–clonic seizures; controlled and resolved after approximately 1 year	None reported
Ataxia/unsteadiness	Recurrent episodes, particularly during illness	Episodes associated with hypotonia, tremors and dysarthria
Dysarthria	Present during episodes	Present during episodes
Eye findings	Astigmatism, strabismus/squint, episodic ptosis	Squint during episodes
MRI findings	Basal ganglia changes with Leigh features	Not available
CNS/metabolic crisis episodes	Frequent; triggered by infection or missed bicarbonate treatment	Recurrent hypotonia and neurological episodes beginning at approximately 2 years

Abbreviations: CNS, central nervous system; MRI, magnetic resonance imaging.

**Table 4:** Metabolic and multisystem complications.

Feature	KU-50.IV-2	KU-50.IV-5
Lactic acidosis	Chronic/intermittent	Present; managed with sodium bicarbonate
Metabolic crises	Frequent; triggered by illness or missed therapy	Episodes of acidosis during crises
Obesity	Morbid obesity (BMI recorded as 60%)	Morbid obesity noted at PICU admission
Diabetes mellitus	Type 2 diabetes mellitus	Not reported
Hypertension	Yes	Not reported
Sleep apnea	Obstructive; BiPAP-dependent	Ventilator-dependent type II respiratory failure
Respiratory complications	Multiple admissions; BiPAP and PICU support	Multiple PICU admissions; tracheostomy and repeated ventilator dependence
Hearing loss	Bilateral sensorineural hearing loss	Not reported

Abbreviations: BiPAP, bilevel positive airway pressure; PICU, pediatric intensive care unit.

**Table 5:** Hospital admissions, major interventions and clinical outcome.

Event	KU-50.IV-2	KU-50.IV-5
PICU admissions	Multiple; respiratory crises and metabolic decompensation	Multiple; respiratory crises, hypercapnia and infections
Mechanical ventilation	BiPAP; intermittent SIMV	Continuous invasive ventilation; tracheostomy
Major infections	Bacterial: MRSA and *Pseudomonas*; viral: parainfluenza 4, EBV and HSV	MRSA and pneumonia; multiple positive cultures from tracheostomy
Ophthalmological intervention	Macular photocoagulation; corrective glasses	Eye discharge; episodic squint
Outcome	Alive at 18 years	Deceased following tracheostomy obstruction; [7 years]

Abbreviations: BiPAP, bilevel positive airway pressure; EBV, Epstein–Barr virus; HSV, herpes simplex virus; MRSA, methicillin-resistant Staphylococcus aureus; PICU, paediatric intensive care unit; SIMV, synchronised intermittent mandatory ventilation.

**Table 6:** Neuroimaging findings in KU-50.IV-2.

MRI Sequence/Feature	Findings	Interpretation
Axial FLAIR	Abnormally increased signal in cortical and subcortical regions of the bilateral anterior frontal lobes (parafalcine) and left insular cortex. Similar altered signal intensities along the bilateral parieto-occipital lobes.	Cortical/subcortical involvement suggestive of metabolic/mitochondrial encephalopathy
ADC maps	No diffusion restriction	No evidence of acute ischaemic change
Axial T1WI	Bilateral low signal intensity in the anterior frontal lobes and left insular cortex	Corresponds to the abnormal FLAIR signal
Axial SWAN	No blooming signal; no haemorrhage or calcification	No evidence of microbleeding or mineral deposition
Coronal STIR	Bilateral high signal intensity in the frontal lobes and left insular cortex	Confirms frontal and insular cortical/subcortical involvement
Neurological correlation	Severe epileptic encephalopathy, Leigh disease, global cognitive impairment and breakthrough seizures	Consistent with significant CNS involvement

Abbreviations: ADC, apparent diffusion coefficient; CNS, central nervous system; FLAIR, fluid-attenuated inversion recovery; STIR, short tau inversion recovery; SWAN, susceptibility-weighted angiography; T1WI, T1-weighted imaging.

**Table 7:** Ophthalmological findings in KU-50.IV-2.

Feature	Finding
Visual acuity	Counting fingers at 50 cm with both eyes open
Fixation and motility	Poor fixation; large-angle left exotropia (~50 prism diopters)
Anterior segment and media	Normal; clear media
Fundoscopy	Bilateral optic disc pallor; macula clinically normal
Macular OCT	OD: Parafoveal and perifoveal thinning (<1st percentile); central subfield within normal limits. OS: not quantifiable due to poor fixation; preserved foveal contour on qualitative B-scan review
Optic nerve head OCT	Not available
MRI (visual pathway)	Reduced volume of both optic nerves and the optic chiasm; bilateral parieto-occipital cortical/subcortical signal abnormality
Final ophthalmological diagnosis:	Cerebral (cortical) visual impairment with concomitant bilateral optic atrophy

Abbreviations: MRI, magnetic resonance imaging; OCT, optical coherence tomography; OD, right eye (oculus dexter); OS, left eye (oculus sinister).

**Table 8:** ACMG/AMP classification of *PDHA1* c.754C>G (p.Leu252Val).

ACMG/AMP Criterion	Evidence in This Study	Strength
PM2	The PDHA1 c.754C>G (p.Leu252Val) variant is absent or extremely rare in population databases, consistent with a rare disease-associated variant.	Supporting
PP1	The variant was present in both affected male siblings and followed the expected X-linked inheritance pattern, with maternal transmission.	Supporting
PP3	Multiple in silico prediction tools, including FATHMM, MutationTaster, MetaLR, and MutPred, supported a deleterious effect.	Supporting
PP4	The affected individuals showed a phenotype consistent with PDHA1-associated PDCD, including neonatal lactic acidosis, neurological involvement, and Leigh neuroimaging abnormalities.	Supporting
PM5	A different pathogenic missense variant affecting the same amino-acid residue has been reported in PDHA1.	Moderate
Final classification	Likely pathogenic	

Abbreviations: ACMG, American College of Medical Genetics and Genomics; AMP, Association for Molecular Pathology; FATHMM, Functional Analysis through Hidden Markov Models; MetaLR, Meta Logistic Regression; PDCD, pyruvate dehydrogenase complex deficiency; PDHA1, pyruvate dehydrogenase E1 alpha 1 subunit; PM, pathogenic moderate evidence; PP, pathogenic supporting evidence.

**Table 9:** ACMG/AMP Classification of *DCLK2* c.101G>A (p.Ser34Asn).

ACMG/AMP Criterion	Evidence in This Study	Strength
PM2	The *DCLK2* c.101G>A (p.Ser34Asn) variant is absent or extremely rare in population databases.	Moderate
PM1	The variant affects a conserved region of DCLK2 with potential functional relevance.	Moderate
PP3	Multiple computational predictors supported a potential damaging effect, including GenoCanyon, likelihood ratio test, PolyPhen-2, and FATHMM-MKL, although predictions were not completely concordant.	Supporting
PP1	The homozygous variant segregated with the affected phenotype in the family and was located within the shared homozygous IBD interval identified in the two affected siblings.	Supporting
PP4	The variant was identified in individuals with a severe neurological phenotype, including Leigh neuroimaging abnormalities and marked visual involvement, in the context of *PDHA1*-associated PDCD.	Supporting
Final interpretation	The genetic, segregation, IBD, and computational findings support *DCLK2* c.101G>A (p.Ser34Asn) as a candidate modifier of the *PDHA1*-associated phenotype.	Candidate modifier of disease severity

Abbreviations: ACMG, American College of Medical Genetics and Genomics; AMP, Association for Molecular Pathology; *DCLK2*, doublecortin-like kinase 2; FATHMM-MKL, Functional Analysis through Hidden Markov Models-Multiple Kernel Learning; IBD, identity by descent; PDCD, pyruvate dehydrogenase complex deficiency; *PDHA1*, pyruvate dehydrogenase E1 alpha 1 subunit; PM, pathogenic moderate evidence; PP, pathogenic supporting evidence.

## Data Availability

Raw sequencing data involves identifiable human genetic information which cannot be publicly released.

## Supplementary Materials

The following supporting information can be downloaded at: https://www.mdpi.com/article/10.3390/jcm15218336/s1, Table S1. Chronological Clinical Course and Major Disease Milestones, including Table S1A. Timeline of Major Clinical Events in KU-50.IV-2 and Table S1B. Timeline of Major Clinical Events in KU-50.IV-5. Supplementary Table S2. Detailed Laboratory and Investigation Findings, including Table S2A. Selected Metabolic and Laboratory Findings Table S2B. Additional Neurological and Neuroimaging Findings, and Table S2C. Detailed Ophthalmological Findings. Supplementary Table S3. Detailed Hospitalization, Respiratory and Treatment History. Table S4. In Silico Prediction Results for *PDHA1* c.754C>G; p.Leu252Val and Table S5. In Silico Prediction Results for *DCLK2* c.101G>A; p.Ser34Asn. Figure S1: Brain MRI-Axial SWAN image demonstrating no blooming signal, with no evidence of haemorrhage or calcification.
