# Supplementary material for: A Homozygous Missense Variant in DCLK2 as a Potential Modifier of Disease Severity in PDHA1-Associated Primary Pyruvate Dehydrogenase Complex Deficiency
Source: J Clin Med. 2026 Oct 26;15(21):8336. doi: 10.3390/jcm15218336 (PMC13764091; doi:10.3390/jcm15218336)
Supplement: Supplementary file 1 [file jcm-15-08336-s001.zip › jcm-4568829-supplementary.pdf]

A Homozygous Missense Variant in *DCLK2* as a Potential Modifier of Disease Severity in *PDHA1*-Associated Primary Pyruvate Dehydrogenase Complex Deficiency

Supplementary Material (9 items: 8 tables and 1 figure)

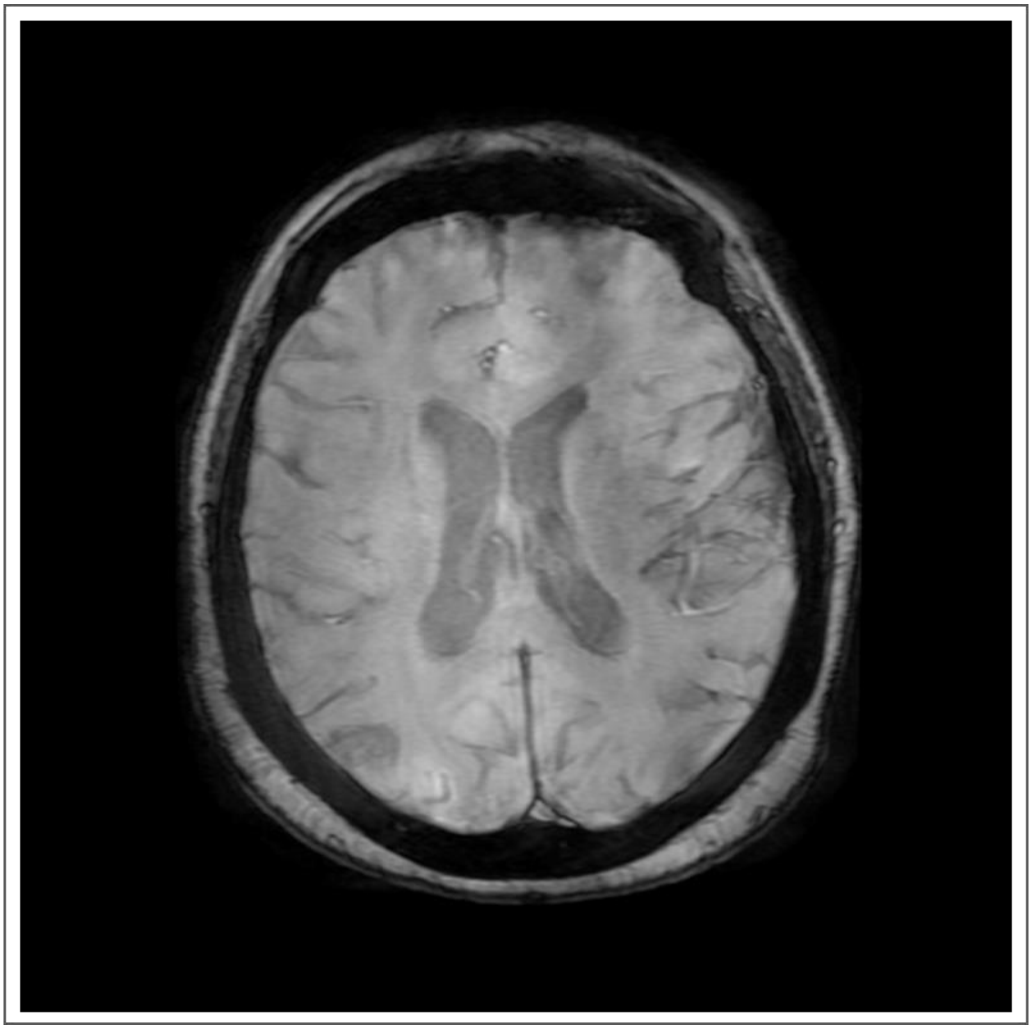

Figure S1: Brain MRI-Axial SWAN image demonstrating no blooming signal, with no evidence of haemorrhage or calcification.

Supplementary Table S1. Chronological Clinical Course and Major Disease Milestones

Table S1A. Timeline of Major Clinical Events in KU-50.IV-2

Age	Major clinical event
Birth	Born preterm at 33 weeks' gestation; NICU admission
5 months	First seizure associated with lactic acidosis
2–3 years	Recurrent encephalopathy and ataxia
6 years	Brain MRI demonstrated basal ganglia involvement
7 years	PICU admission with coma and metabolic acidosis
9 years	Hypercapnic respiratory failure
10 years	Hypertension diagnosed
11 years	Muscle biopsy demonstrated reduced PDH E1-α subunit expression
15 years	Persistent morbid obesity and metabolic instability
19 years	MICU admission with sepsis and respiratory failure

Table S1B. Timeline of Major Clinical Events in KU-50.IV-5

Age	Major clinical event
Birth	Born at term; NICU admission
2 years	Episodic hypotonia and encephalopathy
7 years	Recurrent respiratory failure
7 years	PICU admission with cardiac arrest
7 years	Severe pneumonia
7 years	Medullary involvement followed by tracheostomy
7 years	Recurrent chest infections
7 years	Death following acute tracheostomy obstruction

Supplementary Table S2. Detailed Laboratory and Investigation Findings

Table S2A. Selected Metabolic and Laboratory Findings

Parameter	KU-50.IV-2	KU-50.IV-5
Lactate	Chronic/intermittent elevation	Elevated during metabolic episodes
Acid–base status	Recurrent metabolic acidosis	Recurrent metabolic acidosis
Ammonia	Normal	Normal
PDH enzyme activity	Reduced in muscle	Reduced in fibroblasts

Table S2B. Additional Neurological and Neuroimaging Findings

Investigation/Finding	KU-50.IV-2	KU-50.IV-5
Brain MRI	Basal ganglia involvement with additional cortical/subcortical abnormalities	No detailed MRI assessment available
Brain CT	No significant abnormality documented	No significant abnormality documented
EEG	No persistent epileptiform abnormality documented	No contributory abnormality documented

Table S2C. Detailed Ophthalmological Findings

Finding	KU-50.IV-2	KU-50.IV-5
Visual acuity	Counting fingers at 50 cm in both eyes	Not documented
Ocular motility	Large-angle left exotropia (~50 prism dioptres)	Intermittent squint
Fundoscopy	Bilateral optic disc pallor consistent with optic atrophy	No specific abnormality documented
OCT	Bilateral macular thinning and RNFL thinning, greater on the left	Not performed
Other ophthalmological findings	Earlier retinal/optic disc abnormalities and subsequent ophthalmological intervention	Eye discharge reported
Final ophthalmological assessment	Cortical blindness with secondary bilateral optic atrophy	—

Supplementary Table S3. Detailed Hospitalisation, Respiratory and Treatment History

Clinical feature	KU-50.IV-2	KU-50.IV-5
Intensive care admissions	Recurrent admissions for metabolic decompensation, infection and respiratory failure	Recurrent PICU admissions for respiratory failure, infections and metabolic decompensation
Respiratory support	Intermittent non-invasive and invasive mechanical ventilation; BiPAP support	Prolonged invasive mechanical ventilation and tracheostomy
Major infections	Bacterial and viral infections, including MRSA, Pseudomonas, parainfluenza, EBV and HSV	Recurrent respiratory infections, including MRSA and pneumonia
Metabolic management	Sodium bicarbonate supplementation and mitochondrial cocktail therapy	Supportive metabolic and mitochondrial therapy
Antiepileptic treatment	Lacosamide, brivaracetam and clonazepam	—
Major respiratory complications	Hypercapnic respiratory failure and recurrent respiratory decompensation	Respiratory failure, tracheostomy complications and acute tracheostomy obstruction
Outcome	Alive at late adolescence	Died in childhood following acute tracheostomy obstruction

In Silico Prediction Results for the Identified Variants

Table S4. In Silico Prediction Results for *PDHA1* c.754C>G; p.Leu252Val

Prediction tool	Score	Interpretation
FATHMM	−3.71	Deleterious
MutationTaster	0.999966	Disease-causing
MetaLR	0.583	Predicted damaging
MutPred	0.583	Predicted pathogenic effect
M-CAP	0.225	Below pathogenicity threshold
REVEL	0.280	Low/indeterminate prediction

Table S5. In Silico Prediction Results for *DCLK2* c.101G>A; p.Ser34Asn

Prediction tool	Score	Interpretation
GenoCanyon	0.999999	High probability of functional significance
LRT	0.000202	Deleterious
PolyPhen-2 (HDIV)	0.281–0.799	Possibly to probably damaging
fathmm-MKL (Coding)	0.749	Damaging
MetaSVM	−0.7831	Tolerated
M-CAP	0.0547	Below pathogenicity threshold
